# Regulatory role of local tissue signal Del-1 in cancer and inflammation: a review

**DOI:** 10.1186/s11658-021-00274-9

**Published:** 2021-07-03

**Authors:** Meng Li, Di Zhong, Guozhong Li

**Affiliations:** Department of Neurology, First Affiliated Hospital of Harbin Medical University, No. 23 Youzheng Road, Harbin, 150001 Heilongjiang China

**Keywords:** Del-1, Local tissue signals, Cancer microenvironment, Inflammation resolution, Immunity homeostasis

## Abstract

Developmental endothelial locus-1 (Del-1) is a secretory, multifunctional domain protein. It can bind to integrins and phosphatidylserine. As a local tissue signal, it plays a regulatory role in the cancer microenvironment and inflammation. Del-1 has destructive effects in most cancers and is associated with the progression and invasion of some cancers. In contrast, Del-1 also plays a protective role in inflammation. Del-1 regulates inflammation by regulating the generation of neutrophils in bone marrow, inhibiting the recruitment and migration of neutrophils and accelerating the clearance of neutrophils by macrophages. Del-1 and IL-17 are reciprocally regulated, and their balance maintains immune system homeostasis. Del-1 is expected to become a new therapeutic target for inflammatory disorders such as multiple sclerosis.

## Background

A wide variety of local tissue signals exist in the human body. Local tissues are believed to be passive recipients of immunity and cancer, but recent studies have found that they are active regulators [1]. Local tissue signals can remold immunity and play crucial roles in immune-driven inflammatory diseases and cancer [2]. Some signals exert different functions at different positions. In some circumstances, some local tissue signals can regulate the recruitment and activation of immune cells to control the initiation and termination of the immune response. In others, they can regulate the function and phenotype of local tissues and recruit immune cells. These signals are mainly secreted by local stromal and parenchymal cells, including cytokines, growth factors, antimicrobial peptides, and other locally acting factors [1, 3]. They promote or inhibit the interaction of local tissues with immune cells through direct effects or intercellular adhesion [4, 5]. Local tissue signals are essential in the local tissue microenvironment, and their compartmentalized expression is considered to optimize the spatial regulation of the body.

Developmental endothelial locus-1 (Del-1) is a representative of local tissue signals and exerts different regulatory functions in different expression areas [6]. For instance, Del-1 accelerates the process of inflammation resolution in inflammatory areas, but not in non-inflammatory areas. The functions of many other local tissue signals remain unclear. It is useful to study the role of Del-1 for understanding how local tissue signals regulate the local tissue microenvironment, including how they maintain homeostasis of the immune system, and regulate the invasion of cancer and other unknown functions. In this paper, we present a review of the regulatory role of local tissue signal Del-1 in cancer and inflammation.

## Structure, ligands, and functions of Del-1

Del-1 is a 52 kDa extracellular matrix glycoprotein that is primarily produced by endothelial cells during embryological vascular development [7]. Macrophages, neuronal cells, osteoclasts, and some hematopoietic microenvironment cells can also produce Del-1 [8–10]. Del-1 consists of three N-terminal EGF-like repeats (E1, E2, and E3) and two C-terminal discoidin I-like domains (C1 and C2). EDIL3 (EGF like repeats and discoidin domains 3) is the gene encoding Del-1 [6]. Del-1 not only interacts with αv (αvβ3 and αvβ5) integrins through an RGD motif in the second EGF repeat [11, 12] but also interacts with glycosaminoglycans and phosphatidylserine (PS) through discoidin I-like domains [13]. See the Protein Data Bank (PDB) website for details of Del-1 3D structure (http://www.rcsb.org/structure/4d90). Del-1 can bind to β2 integrins, which have distinct CD11 subunits and a common CD18 subunit [14]. αLβ2 integrin (LFA-1, lymphocyte function-associated antigen 1; CD11a/CD18) mediates the process by which leukocytes adhere firmly to the vascular endothelium and transmigrate through the vessel wall, which results in their recruitment to inflamed tissue [15]. In the vessel lumen, αMβ2 integrin (MAC-1, macrophage-1 antigen; CD11b/CD18) mediates not only the crawling of leukocytes on the endothelium, but also the process by which leukocytes search for a proper site to transmigrate from the vessel [16]. Del-1 can bind to αLβ2 and αMβ2 integrins and prevent them from binding to intercellular adhesion molecule-1 (ICAM-1), thus preventing binding between leukocytes and the endothelium [17]. Del-1 can also bind to αvβ3 integrin on the macrophage at one end and to PS on the apoptotic cell at the other end, thereby acting as a bridge to mediate the efferocytosis of apoptotic cells by macrophages [9, 18]. Genetic knockout of Del-1 has a unique phenotype. In mice with periodontitis, Del-1 deficiency is associated with inflammatory periodontal loss and neutrophil infiltration [19]. In experimental allergic encephalomyelitis (EAE), Del-1 deficiency increases disease severity, increases inflammation and immune cell infiltration in the central nervous system (CNS), increases IL-17 levels, and breaks down the blood–brain barrier (BBB) [8]. In endothelial cells, Del-1 deficiency increases LFA-1 dependent leukocyte adhesion in vitro and in vivo. Del-1 deficient mice display higher neutrophil accumulation during lung inflammation, but this condition can be reversed in Del-1/LFA-1 double-deficient mice [14]. In postoperative peritoneal adhesion (PPA) mice, Del-1 deficiency increases the incidence and severity of PPA, increases acute inflammation, and increases the deposition of extracellular matrix (ECM) proteins in the surgically traumatized peritoneum [20]. In hematopoietic stem cells (HSCs), Del-1 deficiency increases long-term HSC quiescence [21]. In mice with lung fibrosis, Del-1 deficiency activates transforming growth factor β (TGF-β), thereby increasing the production of collagen [22].

## Elevated levels and progression-promoting effects of Del-1 in cancer

Previous studies have shown that under the effect of microenvironmental signals, tumor-related macrophages and leukocytes can differentiate into specific phenotypes to foster tumor progression and suppress adaptive immunity [23]. The growth and metastasis of cancer are associated with angiogenesis, and Del-1 is involved in angiogenesis [24]. In the original site, cancer cells interact with tumor-derived endothelial cells, and in the secondary site, cancer cells interact with normal tissue-derived endothelial cells. Studies have shown that the expression of Del-1 is upregulated in cancer cells; αvβ3, αvβ5, and their ligands Del-1 and L1-CAM (CD171) play essential roles in the process of cancer cell adhesion at the primary site [25, 26]. Since then, researchers have started to focus on the relationship between cancer and Del-1. The relationship between breast cancer and Del-1 has been most widely studied. Researchers examined the level of Del-1 in the plasma and circulating extracellular vesicles (EVs) of early stage breast cancer patients and found that the levels of Del-1 were upregulated both in the plasma and EVs compared to those of the controls. Furthermore, the sensitivity of Del-1 for early stage breast cancer diagnosis was higher than that of CA-153. Therefore, Del-1 in the plasma and EVs may be a sensitive biomarker that can identify early stage breast cancer and distinguish breast cancer from benign breast tumors and noncancerous diseases [27]. In another study, although the expression of Del-1 mRNA was found in all breast cancer cell lines, the rate and intensity were much higher in triple-negative breast cancer (TNBC), and Del-1 was correlated with cancer progression and worse survival trends [28]. Therefore, Del-1 is likely to act as a biomarker and progression predictor in patients with TNBC [29]. One study elucidated that tamoxifen-resistant breast cancer has a strong correlation with Del-1 overexpression, and its progression can be inhibited by Del-1 depletion, which means that the sensitivity of tamoxifen is restored [30]. Therefore, downregulating the level of Del-1 is a potential therapeutic strategy for some types of breast cancer.

**Fig. 1:**
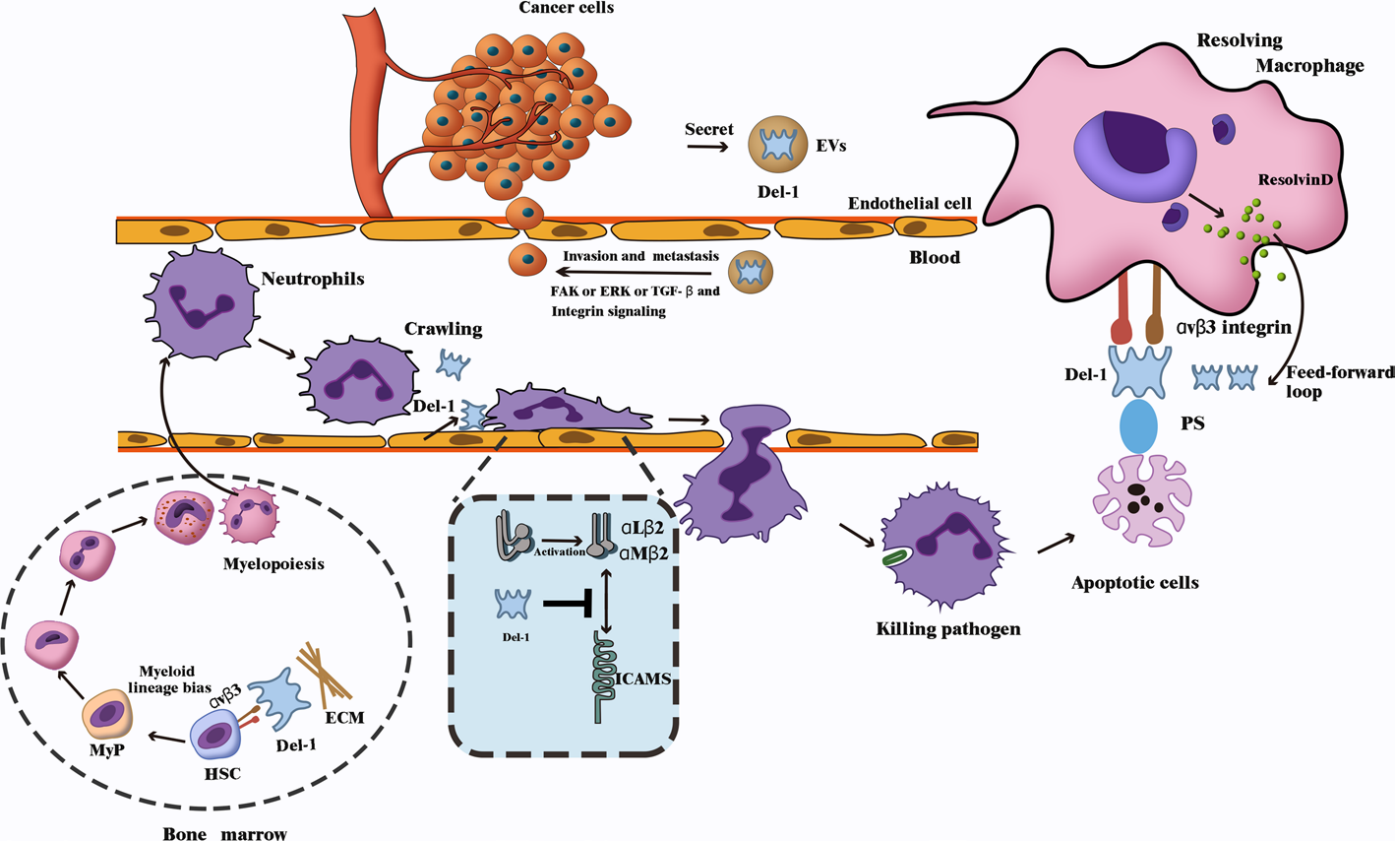
Function of Del-1 in cancer and inflammation. The framed section represents the tumor microenvironment and the unframed section represents inflammatory conditions. (1) Del-1 is correlated with the invasion and metastasis of some cancers through FAK, TGF-β, ERK, or other unknown signaling pathways; (2) Del-1 in the bone marrow niche binds to HSCs αvβ3 integrin on one end and interacts with the extracellular matrix at the other end, thus promoting HSC proliferation and differentiation into the myeloid lineage; (3) In the in-vessel lumen, endothelial cell-derived Del-1 blocks the binding of αLβ2 (LFA-1) integrin to ICAM-1 on vascular endothelial cells, thereby blocking the adhesion and migration of neutrophils. Some studies have found that Del-1 can also block αMβ2 (Mac-1) integrin, which mediates neutrophil crawling in the official cavity; (4) In the course of the resolution of inflammation, neutrophils transform into apoptotic cells after exerting an anti-inflammatory effect. Del-1 connects PS on apoptotic cells at one end, which is an “eat-me” signal, and αvβ3 integrin on macrophages at the other end as a bridge for cell-to-cell communication to promote the uptake of apoptotic cells by macrophages. *Del-1* developmental endothelial locus-1, *EVs* extracellular vesicles, *HSCs* hematopoietic stem cells, *MyP* myeloid progenitors, *ECM* extracellular matrix, *ICAM* intercellular adhesion molecule, *PS* phosphatidylserine

In addition to breast cancer, EDIL3 expression increases in hepatocellular carcinoma and predicts a poor prognosis [31]. It also enhances the tumorigenic, metastatic, and angiogenic potential through TGF-β and ERK signaling in hepatocellular carcinoma [32]. In addition, it is increased in endometrial, colon, bladder, and pancreatic cancers [33–36]. Del-1 appears to be negatively correlated with some types of cancers. Del-1 mRNA expression is downregulated in human lung adenocarcinoma cell lines [37], although it is related to angiogenesis, mesenchymal phenotype, and progression of lung adenocarcinoma [38]. Mechanistically, Del-1 suppresses NF-κB-activated macrophage migration inhibitory factor production in macrophages and may be a treatment target in some chronic inflammation-associated cancers [39] (Fig. 1).

In conclusion, Del-1 plays a destructive role in most cancers, but some results are controversial, and more experiments are needed to elucidate its functions in cancer.

## Del-1 is a regulator of inflammation

### Del-1 promotes proliferation and differentiation of HSCs into the myeloid lineage

Peripheral inflammation or infection triggers myeloid cell formation in the bone marrow, which is essential for replenishing consumed peripheral neutrophils during infection or inflammation. This process, which includes the feedback loop between the peripheral blood and bone marrow, is known as emergency myelopoiesis [40–42]. Emergency myelopoiesis is an important homeostatic mechanism in immunity and inflammation. Del-1 promotes emergency myelopoiesis, and it is also a component of the hematopoietic microenvironment of the bone marrow that is called the niche [21]. The niche supports the survival and renewal of hematopoietic stem cells (HSCs), maintains their quiescence, prevents aging caused by DNA, and promotes replication of HSCs [43]. Arteriolar endothelial cells, osteoblastic lineage cells, and a specialized perivascular mesenchymal stromal cell type, also known as CXCL-12-abundant reticular (CAR) cells, in the bone marrow niche can produce Del-1[21].

Studies have found that Del-1 regulates HSC proliferation and differentiation toward the myeloid lineage. In Del-1 deficient mice, G-CSF-dependent hematopoiesis and myeloid progenitor proliferation are dramatically reduced. It is speculated that Del-1 is the key substance involved in the differentiation of HSCs into the myeloid lineage [21]. Excessive expression of Del-1 in the niche can increase the cell diversity of the bone marrow and promote the reconstitution of the myeloid lineage [44].

Del-1 regulates the process of myelopoiesis as follows: in the bone marrow niche, Del-1 binds to HSC αvβ3 integrin on one end and interacts with the extracellular matrix at the other end, thus promoting HSC proliferation and differentiation into the myeloid lineage.

### Del-1 exerts anti-inflammatory effects by inhibiting neutrophil recruitment and migration

Del-1 levels in the brain are significantly higher than those in the gingival and lung tissues, which is consistent with the previous view that the brain is an immune-privileged organ [14]. Del-1 deficiency in EAE mice results in the overproduction of IL-17 and the migration of neutrophils to the spinal cord, accompanied by demyelination and axonal loss, resulting in more severe damage to the BBB [8]. IL-17 can promote the migration of neutrophils to the spinal cord, aggravating damage to the BBB and increasing endothelial permeability. Therefore, it is speculated that Del-1 can affect the migration of neutrophils and regulate immune processes in the body.

The mechanisms by which Del-1 regulates neutrophil recruitment and migration are as follows. In the vessel lumen, endothelial cell-derived Del-1 blocks the binding of αLβ2 (LFA-1) integrin to ICAM-1 on vascular endothelial cells, thereby blocking the adhesion and migration of neutrophils. Some studies have found that Del-1 can also block αMβ2 (Mac-1) integrin, which mediates neutrophil migration in the lumen. Whether Del-1 directly inhibits neutrophil crawling has not been fully confirmed [16] (Fig. 1).

### Del-1 accelerates macrophage efferocytosis and inflammation resolution

Previous studies have confirmed that IL-17 levels represent the severity of inflammation, as IL-17 promotes the progression of inflammation [45]. Del-1 and IL-17 negatively regulate each other. Therefore, Del-1 may inhibit the progression of inflammation and promote its resolution. The levels of Del-1 in the gingival crevicular fluid increase in patients with periodontitis after scaling and root planning, suggesting that Del-1 promotes the resolution of inflammation [9]. The expression of Del-1 in patients with multiple sclerosis decreases in the chronic active phase but does not decrease in the inactive phase, suggesting that the lack or low levels of Del-1 may lead to disease progression [8]. Resolvin D1 (RvD1) is a member of a family of pro-resolving lipid mediators, and an endogenous lipid mediator metabolized by docosahexaenoic acid (DHA) that promotes the resolution of inflammation [46]. Recently, researchers have found that in the process of inflammatory resolution, pro-resolving phenotype macrophages can secrete RvD1, RvD1 interacts with the G-protein-coupled receptor 32 (GPR32) and the lipoxin A4 receptor/formylpeptide receptor-2 (ALX/FPR2) on the endothelial cell membrane, promoting the phosphatidylinositol 3-kinase (PI3K)/Akt signaling pathway in the cytoplasm, and inhibiting glycogen synthase kinase 3β (GSK3β), thereby promoting binding of the transcription factor CCAAT enhancer-binding protein β (C/EBPβ) to the promoter of EDIL3 and the expression of Del-1 [47]. The positive regulatory effect of RvD1 on Del-1 suggests that Del-1 may play a role in the resolution of inflammation.

The mechanisms by which Del-1 regulates macrophage efferocytosis and inflammation resolution are as follows: during the resolution of inflammation, neutrophils transform into apoptotic cells after exerting their inflammatory effects [48]. Del-1 connects PS on the apoptotic cells at one end, which is an “eat-me” signal, and connects αvβ3 integrin on the macrophages at the other end, as a bridge for cell-to-cell communication and the uptake of apoptotic cells by macrophages. Meanwhile, Del-1 causes the hepatic X receptor-related pathway to reprogram macrophages, which transforms them into a pro-resolving phenotype, thereby upregulating transforming growth factor β (TGFβ), resolvins, and other pro-resolving factors. RvD1 can promote the expression of Del-1, thus forming a positive feedback loop to accelerate the resolution of inflammation [9] (Fig. 1).

### Del-1 inhibits osteoclastogenesis and protects against inflammatory bone loss

In periodontitis, the mechanism of osteoclastogenesis can be roughly divided into the following categories: RANKL-dependent osteoclastogenesis, partial RANKL-dependent osteoclastogenesis initiated by LPS/TLR signaling, and other regulatory pathways [49]. Nuclear factor of activated T-cells (NFATc1) is a crucial regulator of osteoclastogenesis. Del-1 inhibits expression of NFATc1 in a Mac-1 integrin-dependent manner in periodontitis. Del-1 regulates mouse osteoclast differentiation and function, and the RGD motif and E1–E3 repeats of Del-1 are involved in osteoclast formation and function. Del-1 also protects against inflammatory bone loss in periodontitis mice, in which Del-1–Fc is a crucial component [10]. The pro-oncogenic and anti-inflammatory roles of Del-1 are summarized in Table 1, based on the original articles listed.

**Table 1:** Roles of Del-1 in disease and pathophysiological conditions

	Disease or pathophysiological conditions	Roles of Del-1	Outcomes	Articles
Pro-oncogene s roles	Liver cancer	Plays an important role in the process of tumor cells moving from the primary site	The expression of Del-1 was higher in the human liver hepatocellular carcinoma cell line, tumor cell-EC adhesion was inhibited by antibodies against αvβ3, αvβ5	Niu et al. [26]
	Breast cancer	A biomarker and a progression predictor	Del-1 are upregulated both in plasma and circulating extracellular vesicles of early-stage breast cancer patients, the sensitivity of Del-1 for early-stage breast cancer diagnosis is higher than in CA-153; Del-1 is correlated with cancer progression and worse survival trend in triple-negative breast cancer	Moon et al. [27], Lee et al. [28]
	Liver cancer	A poor prognosis predictor	EDIL3 was highly expressed in the HCC patients, Multivariate Cox’s analysis showed that the EDIL3 expression level was a significant and independent prognostic parameter for the overall survival rate of HCC patients	Sun et al. [31]
	Liver cancer	Enhancing the tumorigenic, metastatic, and angiogenesis potential	Overexpression of EDIL3, which was regulated by the downregulation of miR-137 in HCC, triggered the activation of ERK and TGF-β signaling through interactions with αvβ3 integrin. Blocking ERK and TGF-β signaling overcomes EDIL3 induced angiogenesis and invasion	Xia et al. [32]
	Bone marrow niche	Promoting the proliferation and differentiation of HSCs into myeloid lineage	In the bone marrow niche, Del-1 binds to HSCs αvβ3 integrin on one end and interacts with the extracellular matrix on the other end, thus promoting HSCs proliferation and differentiation into the myeloid lineage	Mitroulis et al. [21], Chen et al.
Anti-inflammatory roles	Inflammation	Exerting anti-inflammatory effects by inhibiting neutrophil recruitment and migration	In the vessel lumen, endothelial cell-derived Del-1 blocks the binding of αLβ2(LFA-1) integrin on neutrophils to ICAM-1 on vascular endothelial cells, thereby blocking the adhesion and migration of neutrophils	Vestweber et al. [16]
	Inflammation resolution	Accelerating macrophage efferocytosis and inflammation resolution	In the course of the resolution of inflammation, Del-1 connects PS on apoptotic cells at one end to αvβ3 integrin on macrophages at the other end as a bridge of cell-to-cell communication and the uptake of apoptotic cells by macrophages. Del-1 makes the hepatic X receptor-related pathway reprogram macrophages, which transform macrophages into the pro-resolving phenotype	Kourtzelis et al. [9]
	Periodontitis	Inhibits osteoclastogenesis and protects against inflammatory bone loss	Del-1 inhibited the expression of NFATc1 in a Mac-1 integrin-dependent manner in periodontitis. Del-1 regulated mouse osteoclast differentiation and function, RGD motif, and E1–E3 repeats of Del-1 involved in osteoclast formation and function. Del-1 also protected against inflammatory bone loss in periodontitis mice which Del-1-Fc was a crucial component of	Shin et al. [50]

*HSCs* hematopoietic stem cells, *Del-1* Developmental endothelial locus-1, *ICAM-1* intercellular adhesion molecule-1, *TGFβ* transforming growth factor β, *HCC* hepatocellular carcinoma

### Interaction and homeostasis between Del-1 and IL-17

Studies have shown that the progression of periodontitis is controlled in mice doubly deficient in Del-1 and the IL-17 receptor. In contrast, progress has been observed in mice deficient in Del-1. This implies that inflammation caused by Del-1 deficiency is not only due to excessive αLβ2 integrin-mediated neutrophil migration but also due to IL-17 receptor signaling [19]. Next, we revealed that Del-1 and IL-17 antagonize each other in function as well as inhibiting each other’s expression. IL-17 can directly inhibit the expression of Del-1 through the GSK-3β-C/EBPβ pathway. Th17 cells secrete IL-17A, which binds to the IL-17A receptor on endothelial cells, and the kinase GSK3β downstream of the IL-17A receptor in the cytoplasm is activated, after which GSK3β inhibits the binding of the transcription factor C/EBPβ and EDIL3 promoter, thereby inhibiting the expression of Del-1 [47]. However, the mechanism by which Del-1 inhibits IL-17 production has not yet been clarified.

The balance between Del-1 and IL-17 is crucial for maintaining the homeostasis of the immune system. In the stable state, Del-1 and IL-17 inhibit each other and maintain neutrophil recruitment in a balanced state, thus maintaining immune system homeostasis. The expression of Del-1 decreases with age, which is a result of immune aging. Studies have found that chronic inflammation is associated with immune aging [50–53]. In the aging or inflammatory state, IL-17 production increases, and the balance is tilted toward IL-17. IL-17 further directly reduces the expression of Del-1, thereby enhancing neutrophil recruitment and inflammatory bone loss [50]. In the absence or decrease of Del-1, the body is susceptible to IL-17-mediated inflammatory diseases and disease progression.

### Del-1 is a potential therapeutic target in multiple sclerosis and other inflammatory disorders

Del-1 deficiency can increase the severity of disease in patients with EAE and multiple sclerosis. Researchers have conducted experiments on relapsing–remitting EAE mice: After the first clinical attack, mice in the experimental group were given a whole-body injection of the Del-1-Fc segment, while mice in the control group were given the Del-1 block Fc segment. Compared to the control group, clinical relapse in the experimental group was prevented [8]. Thus, Del-1 may be a potential therapeutic target in multiple sclerosis. Del-1-Fc also shows an anti-inflammatory effect in the peripheral blood in periodontitis [19]. EDIL3, the gene encoding Del-1, is believed to be a susceptibility gene for multiple sclerosis and Alzheimer’s disease [54, 55]. Recent studies have found that it is also related to ankylosing spondylitis [56], which is triggered by dysregulation of the IL-23/IL-17 pathway [57]. The role of Del-1 in this process is still unclear. Overall, Del-1 plays a role in multiple sclerosis, ankylosing spondylitis, allergic asthma, peritoneal adhesion, and other inflammatory diseases mediated by IL-17 [20, 58, 59]. Del-1 may be a potential therapeutic target for inflammatory diseases mediated by IL-17.

## Conclusions

Del-1 is a secreted multifunctional protein that has been discovered in recent years, and various studies have identified its coding genes and domains, etc. As a local tissue signal, its function in the cancer microenvironment and local inflammatory tissue is gradually being clarified. Del-1 is related to the progression and invasion of some cancers and plays a destructive role in most cancers, although the results are controversial. Contrary to cancer, the available evidence supports the protective role of Del-1 in inflammation. Del-1 inhibits inflammation mainly by regulating the entire function of neutrophils in inflammation. Del-1 and IL-17 negatively regulate each other and antagonize each other’s function. The balance between them maintains the homeostasis of the immune system. In the future, Del-1 is expected to become a therapeutic target for inflammatory disorders mediated by IL-17, such as multiple sclerosis, psoriasis, asthma, and pulmonary infectious diseases. However, we still do not know whether regulating the expression of Del-1 will activate some cancer-related biological processes in patients with inflammatory disorders or will increase the progression and invasion in cancer patients, or whether Del-1 also interacts with other immune cells and substances. In recent years, knowing many new advances in biological sciences including "omic" screens and gene editing technology, researchers have more ways to study the function of proteins and their interactions. Sequencing technology can analyze the expression changes of EDIL3 in different physiological and pathological conditions. The main goal of post-genomic biology is to construct and predict the complex network of proteins, DNA, RNA and small chemical molecules interacting with Del-1 [60]. CRISPR/cas9 based gene editing technology is developing at an unprecedented speed. Some researchers have used this technology to inhibit inflammatory cytokine receptors and change the response of cells to an inflammatory environment and treat chronic pain [61]. Gene editing technology is also expected to be used to regulate the local tissue signals associated with Del-1, so as to change the tumor immune microenvironment or regulate the immune microenvironment of inflammatory diseases. These aspects may be new directions for future research.

## Data Availability

Not applicable.

## Abbreviations

ALX/FPR2: Lipoxin A4 receptor/formylpeptide receptor-2
BBB: Blood–brain barrier
CAR: CXCL-12-abundant reticular
CNS: Central nervous system
C/EBPβ: CCAAT enhancer-binding protein β
Del-1: Developmental endothelial locus-1
DHA: Docosahexaenoic acid
EAE: Experimental allergic encephalomyelitis
ECM: Extracellular matrix
EDIL3: EGF like repeats and discoidin domains 3
EVs: Circulating extracellular vesicles
GPR32: G-protein-coupled receptor 32
GSK3β: Glycogen synthase kinase 3β
HSCs: Hematopoietic stem cells
ICAM-1: Intercellular adhesion molecule-1
PI3K: Phosphatidylinositol 3-kinase
PPA: Postoperative peritoneal adhesions
PS: Phosphatidylserine
RvD1: Resolvin D1
TGF-β: Transforming growth factor β
TNBC: Triple-negative breast cancer
